# Identification of interneurons required for the aversive response of *Caenorhabditis elegans* to graphene oxide

**DOI:** 10.1186/s12951-018-0373-y

**Published:** 2018-04-27

**Authors:** Guosheng Xiao, He Chen, Natalia Krasteva, Qizhan Liu, Dayong Wang

**Affiliations:** 1 College of Biology and Food Engineering, Chongqing Three Gorges University, Wanzhou, 404100 China; 2 Key Laboratory of Environmental Medicine Engineering in Ministry of Education, Medical School, Southeast University, Nanjing, 210009 China; 3 Institute of Biophysics and Biomedical Engineering, Bulgarian Academy of Science, Sofia, 1113 Bulgaria; 4 School of Public Health, Nanjing Medical University, Nanjing, 211166 China

**Keywords:** Graphene oxide, NLG-1/neuroligin, Aversive behavior, Interneurons, *Caenorhabditis elegans*

## Abstract

**Background:**

So far, how the animals evade the environmental nanomaterials is still largely unclear. In this study, we employed in vivo assay system of *Caenorhabditis elegans* to investigate the aversive behavior of nematodes to graphene oxide (GO) and the underlying neuronal basis.

**Results:**

In this assay model, we detected the significant aversive behavior of nematodes to GO at concentrations more than 50 mg/L. Loss-of-function mutation of *nlg*-*1* encoding a neuroligin with the function in connecting pre- and post-synaptic neurons suppressed the aversive behavior of nematodes to GO. Moreover, based on the neuron-specific activity assay, we found that the NLG-1 activity in AIY or AIB interneurons was required for the regulation of aversive behavior to GO. The neuron-specific activities of NLG-1 in AIY or AIB interneurons were also required for the regulation of GO toxicity.

**Conclusions:**

Using *nlg*-*1* mutant as a genetic tool, we identified the AIY and AIB interneurons required for the regulation of aversive behavior to GO. Our results provide an important neuronal basis for the aversive response of animals to environmental nanomaterials.

**Electronic supplementary material:**

The online version of this article (10.1186/s12951-018-0373-y) contains supplementary material, which is available to authorized users.

## Background

With the rapid increase in nanotechnology, a larger amount of engineered nanomaterials (ENMs) have been generated for industrial and medical applications. Among these ENMs, graphene nanomaterials have attracted massive attention due to their unique mechanical, electronic, and thermal properties [1, 2]. Graphene oxide (GO) is a member of graphene nanomaterials, and can be potentially used in biomedicine, biosensor, and environmental remediation [3–6]. Meanwhile, some evidence from in vivo studies in mammals has demonstrated the potential of GO in inducing pulmonary or reproductive toxicity [7–9]. More recently, it has been further demonstrated that GO could cause the neurotoxicity on zebrafish [10]. In contrast, how the animals evade the GO particles is still largely unclear.

The classic model animal of *Caenorhabditis elegans* has already been widely used in the toxicological study [11, 12]. Using *C. elegans* as an in vivo assay system, it has been shown that GO could result in toxicity on the functions of both primary targeted organs, such as intestine, and secondary targeted organs, such as reproductive organs [13–17]. *C. elegans* is also a wonderful animal model for the study of neurotoxicity of certain toxicants [11, 18, 19]. In *C. elegans*, GO exposure has also been found to be neurotoxic for animals [20, 21]. Additionally, neuronal ERK- or neuroligin/NLG-1-mediated molecular signaling regulated the formation of GO toxicity in nematodes [22, 23].

In organisms, the postsynaptic cell adhesion proteins, such as the neuroligins, act as central organizing molecules to connect pre- and post-synaptic neurons by binding to presynaptic proteins, like the neurexins [24, 25]. In *C. elegans*, the single neuroligin gene is *nlg*-*1*. In this study, we first investigated the aversive behavior of nematodes to GO. Interneurons (also called connector neurons) establish the link between sensory neurons and motor neurons to enable the neuronal communication [26, 27]. Moreover, using *nlg*-*1* mutant as a genetic tool, we identified the interneurons required for the response of nematodes to GO exposure. Our data suggest the crucial role of AIY and AIB interneurons in the regulation of aversive response of nematodes to GO. Our results provide an important basis for the further elucidation of neuronal circuit for the response of nematodes to GO exposure.

## Methods

### Preparation and characterization of GO

**Fig. 1:**
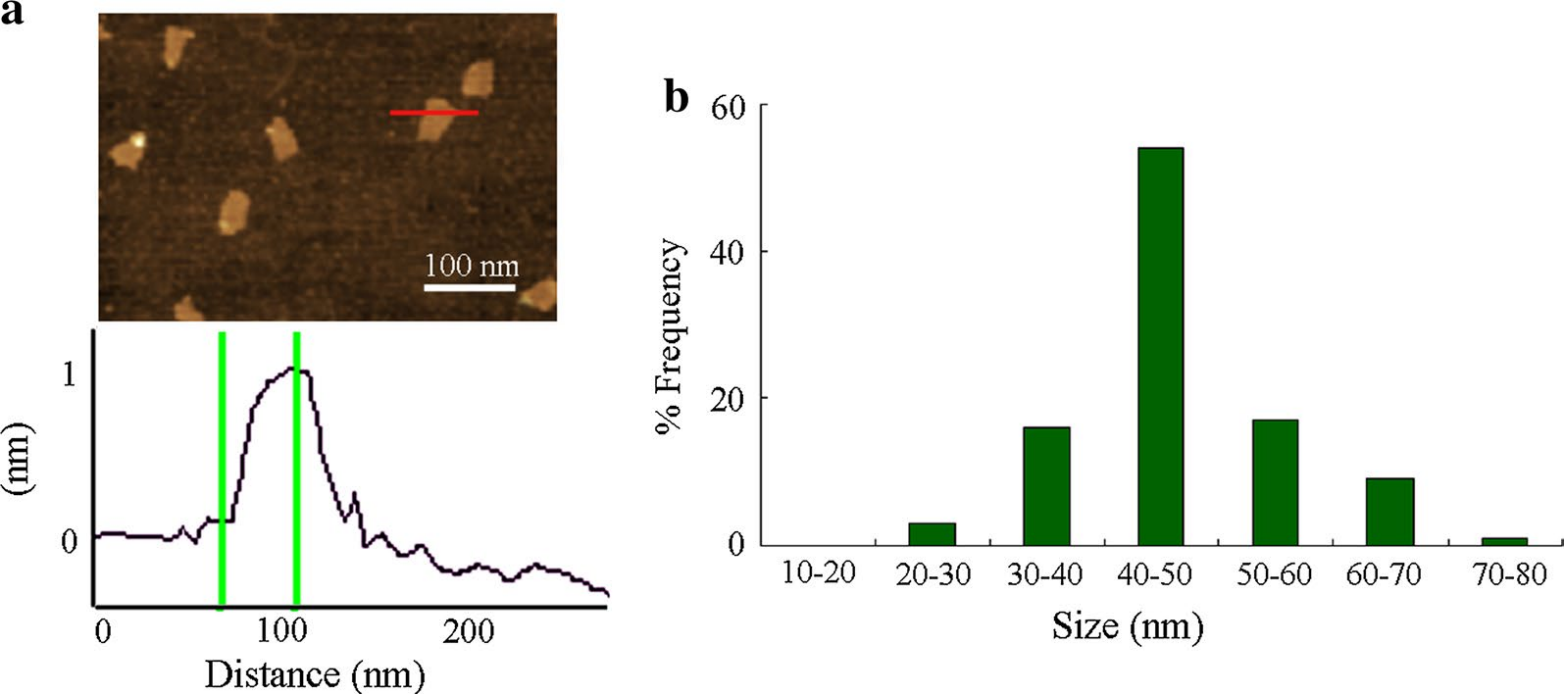
Physiochemical properties of GO. **a** AFM analysis of GO. **b** Size distribution of GO after sonication based on the analysis of Nano Zetasizer

GO was prepared from natural graphite powder based on the modified Hummer’s method [28]. GO was finally obtained by ultrasonication of the as-made graphite oxide. Based on analysis of atomic force microscopy (AFM, SPM-9600, Shimadzu, Japan), the thickness of GO was approximately 1.0 nm in the topographic height, corresponding to one layer property (Fig. 1a). After sonication (40 kHz, 100 W, 30 min), sizes of most of the GO were in the range of 40–50 nm based on the analysis of Nano Zetasizer (Malvern Instrument Ltd., Malvern, UK) (Fig. 1a, b). GO showed the typical G band and D band in Raman spectroscopy [29]. The zeta potential of GO (100 mg/L) in K-medium was − 21.5 ± 2.6 mV [29].

### *C. elegans* strains

The used nematode strains were wild-type N2, mutants of *nlg*-*1*(*tm474*) and *nlg*-*1*(*ok259*), and transgenic strains of *nlg*-*1*(*ok259*)*Ex*(P*ttx*-*3*-*nlg*-*1*), *nlg*-*1*(*ok259*)*Ex*(P*gcy*-*28.d*-*nlg*-*1*), *nlg*-*1*(*ok259*)*Ex*(P*npr*-*9*-*nlg*-*1*), and *nlg*-*1*(*ok259*)*Ex*(P*unc*-*86*-*nlg*-*1*). Both *nlg*-*1*(*tm474*) and *nlg*-*1*(*ok259*) are loss-of-function mutants. Some of the strains were obtained from *Caenorhabditis* Genetics Center. Gravid hermaphrodite nematodes were maintained on normal nematode growth medium (NGM) plates seeded with *Escherichia coli* OP50 at 20 °C as described [30]. The gravid hermaphrodite nematodes were lysed with a bleaching mixture (0.45 M NaOH, 2% HOCl) in order to separate the eggs and the animals. Age synchronous L1-larvae or L4-larvae populations were prepared as described [31].

### Aversive response to GO

**Fig. 2:**
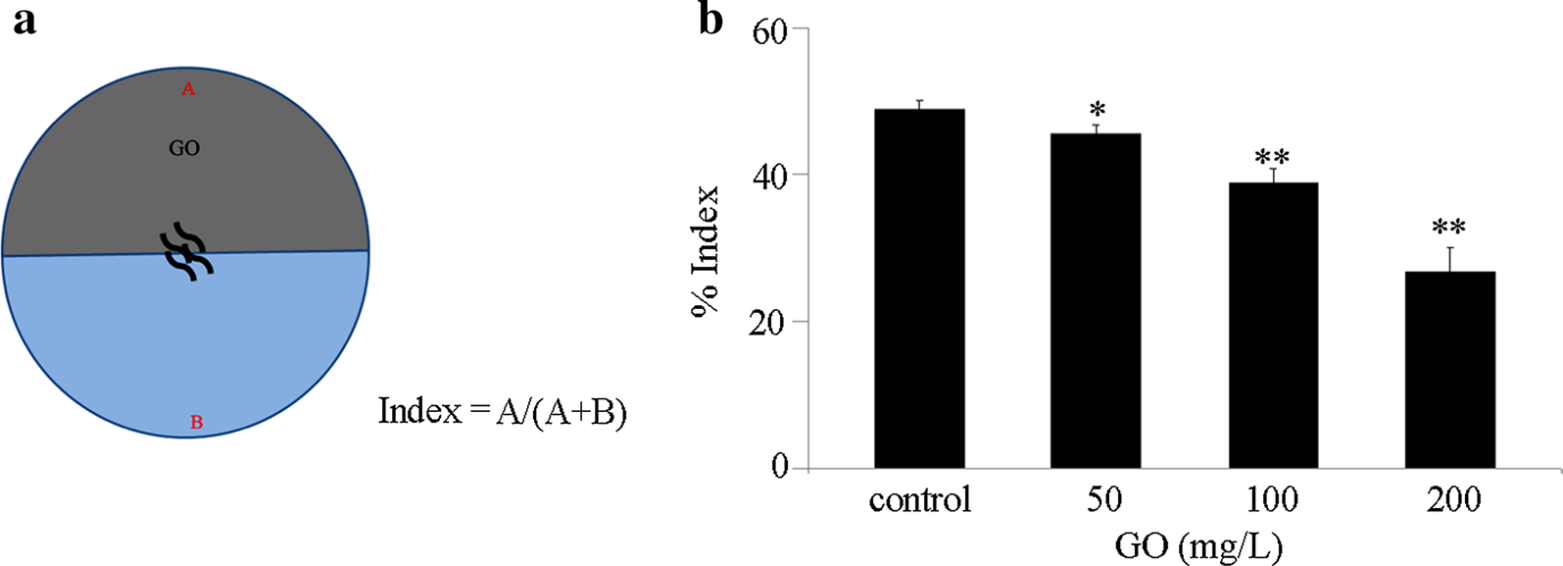
Aversive behavior of wild-type nematodes to GO. **a** Assay model for aversive behavior of nematodes to GO. **b** Aversive behavior of wild-type nematodes to GO at different concentrations. Control, without GO treatment. Bars represent mean ± SD. *P < 0.05 vs control, **P < 0.01 vs control

GO at the used working concentrations (50, 100, and 200 mg/L) was prepared by diluting stock solution (1 mg/mL) with K medium. Before the treatment, GO solutions were sonicated for 30 min (40 kHz, 100 W). To evaluate the aversive responses of nematodes to GO, half of the surface of a 6 cm diameter assay NGM plate was added with GO solution at different concentrations (region A). And then, the examined L4-larvae stage nematodes were placed at the center of the assay NGM plate. After 90 min treatment, the animals on the region A and on the opposite side (region B) were counted, respectively (Fig. 2a). The animals in the middle of the surface of assay NGM plate were omitted. The aversive response of nematodes to GO was evaluated by the percentage of A/(A + B) (Fig. 2a). Forty nematodes were examined per treatment, and ten replicates were performed.

### Toxicity assessment of GO

In nematodes, prolonged exposure (from L1-larvae to young adults) to GO at concentrations more than 0.5 mg/L could cause the decrease in locomotion behavior and the induction of intestinal reactive oxygen species (ROS) production [14]. The used working solution (10 mg/L) was prepaed by diluting stock solution (1 mg/mL) with K medium. Before the exposure, GO solution was sonicated for 30 min (40 kHz, 100 W). Prolonged exposure to GO was performed from L1-larvae to young adults in 12-well sterile tissue culture plates at 20 °C in the presence of food (OP50). After prolonged exposure, the GO exposed nematodes were used for the toxicity assessment using intestinal ROS production and locomotion behavior as the endpoints.

Intestinal ROS production can be used to reflect the functional state of intestine [32]. ROS production was analyzed as described previously [33, 34]. The nematodes were transferred to 1 μM 5′,6′-chloromethyl-2′,7′-dichlorodihydro-fluorescein diacetate (CM-H2DCFDA) to incubate for 3 h at 20 °C in the dark. The examined nematodes were examined at 488 nm of excitation wavelength and 510 nm of emission filter under a laser scanning confocal microscope (Leica, TCS SP2, Bensheim, Germany). Relative fluorescence intensity in intestine was semi-quantified, and the semi-quantified ROS was expressed as relative fluorescence units (RFU) and normalized to the autofluorescence. Fifty nematodes were examined per treatment.

Head thrash and body bend were used to reflect the locomotion behavior [35]. These endpoints were analyzed under dissecting microscope as described [36, 37]. A head thrash is defined as a change in the direction of bending at the mid body, and a body bend is defined as a change in the direction of the part of the nematodes corresponding to the posterior bulb of the pharynx along the *y* axis, assuming that nematode was traveling along the *x* axis. Fifty nematodes were examined per treatment.

### DNA constructs and germline transformation

Promoter region for *ttx*-*3* gene specially expressed in AIY interneurons, *gcy*-*28.d* gene specially expressed in AIA interneurons, *npr*-*9* gene specially expressed in AIB interneurons, or *unc*-*86* gene expressed in AIZ interneurons, was amplified by PCR from wild-type *C. elegans* genomic DNA. These promoter fragments were inserted into pPD95_77 vector in the sense orientation. *nlg*-*1/C40C9.5e* cDNA was amplified by polymerase chain reaction (PCR), and inserted into corresponding entry vector carrying the *ttx*-*3*, *gcy*-*28.d*, *npr*-*9*, or *unc*-*86* promoter sequence. Germline transformation was performed as described by coinjecting testing DNA at the concentration of 10–40 μg/mL and marker DNA of P*dop*-*1::rfp* at the concentration of 60 μg/mL into the gonad of nematodes [38]. The related primer information for DNA constructs is shown in Additional file 1: Table S1.

### Statistical analysis

Data in this article were expressed as mean ± standard deviation (SD). Statistical analysis was performed using SPSS 12.0 software (SPSS Inc., Chicago, USA). Differences between groups were determined using analysis of variance (ANOVA), and probability levels of 0.05 and 0.01 were considered statistically significant.

## Results

### Aversive behavior of wild-type nematodes to GO

On normal NGM plates without the addition of GO, the wild-type nematodes will run randomly, and would be distributed equally on the surface of NGM plates (Fig. 2b). In the aversive behavior assay model, we observed the significant aversive behavior of wild-type nematodes to GO at concentrations of 100 or 200 mg/L after 90 min treatment (Fig. 2b). We also detected the moderate but significant aversive behavior of wild-type nematodes to GO at the concentration of 50 mg/L after 90 min treatment (Fig. 2b). In contrast, after 90 min treatment, we did not observe the obvious aversive behavior of wild-type nematodes to GO at concentrations less than 50 mg/L (data not shown).

### *nlg*-*1* mutation suppressed the aversive behavior of nematodes to GO

**Fig. 3:**
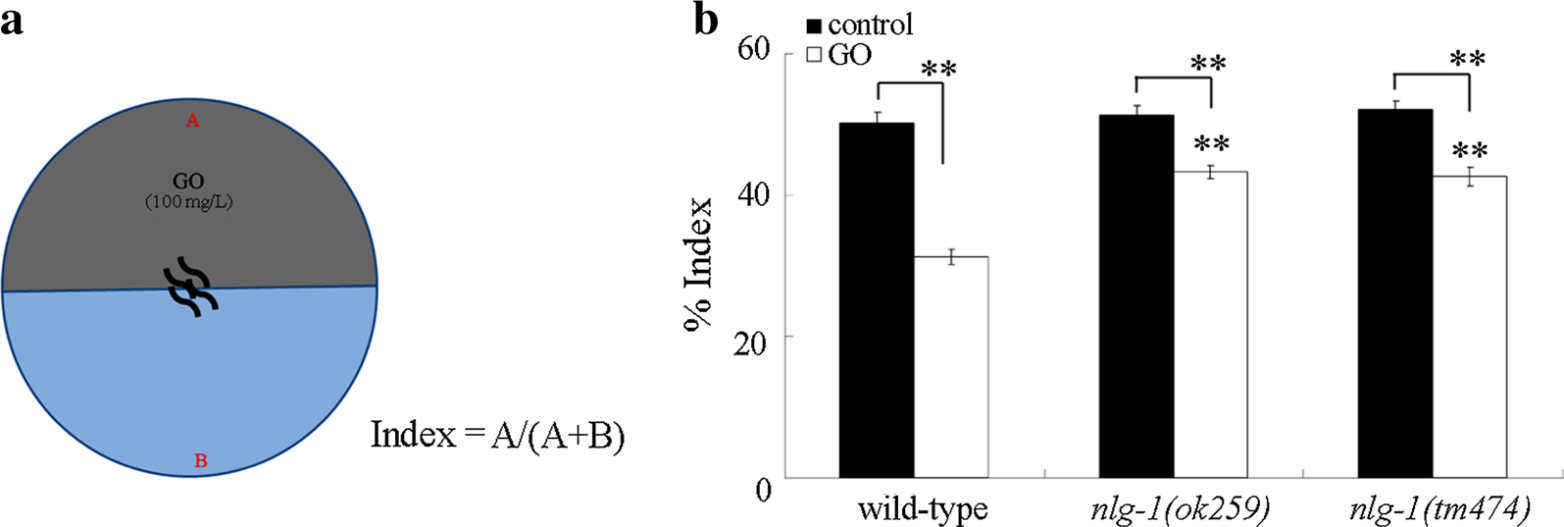
Effect of *nlg*-*1* mutation on aversive behavior of nematodes to GO. **a** Assay model for aversive behavior of nematodes to GO (100 mg/L). **b** Effect of *nlg*-*1* mutation on aversive behavior of nematodes to GO. Control, without GO treatment. Bars represent mean ± SD. **P < 0.01 vs wild-type (if not specially indicated)

Considering the important function of neuroligins in connecting pre- and post-synaptic neurons [24, 25], we next examined the effect of *nlg*-*1* mutation on aversive behavior of nematodes to GO. We focused on the analysis of aversive behavior of nematodes to GO at the concentration of 100 mg/L (Fig. 3a). On normal NGM plates, both wild-type and *nlg*-*1* mutant (*nlg*-*1*(*ok259*) or *nlg*-*1*(*tm474*)) nematodes were observed to be distributed equally on the surface of NGM plates (Fig. 3b). In the aversive behavior assay model, both *nlg*-*1*(*ok259*) mutant and *nlg*-*1*(*tm474*) mutant showed the increased index for assessing aversive behavior to GO (100 mg/L) compared with wild-type nematodes after 90 min treatment (Fig. 3b). Therefore, *nlg*-*1* mutation may suppress the aversive behavior of nematodes to GO in nematodes.

### Neuron-specific activity of NLG-1 in the regulation of aversive behavior of nematodes to GO

**Fig. 4:**
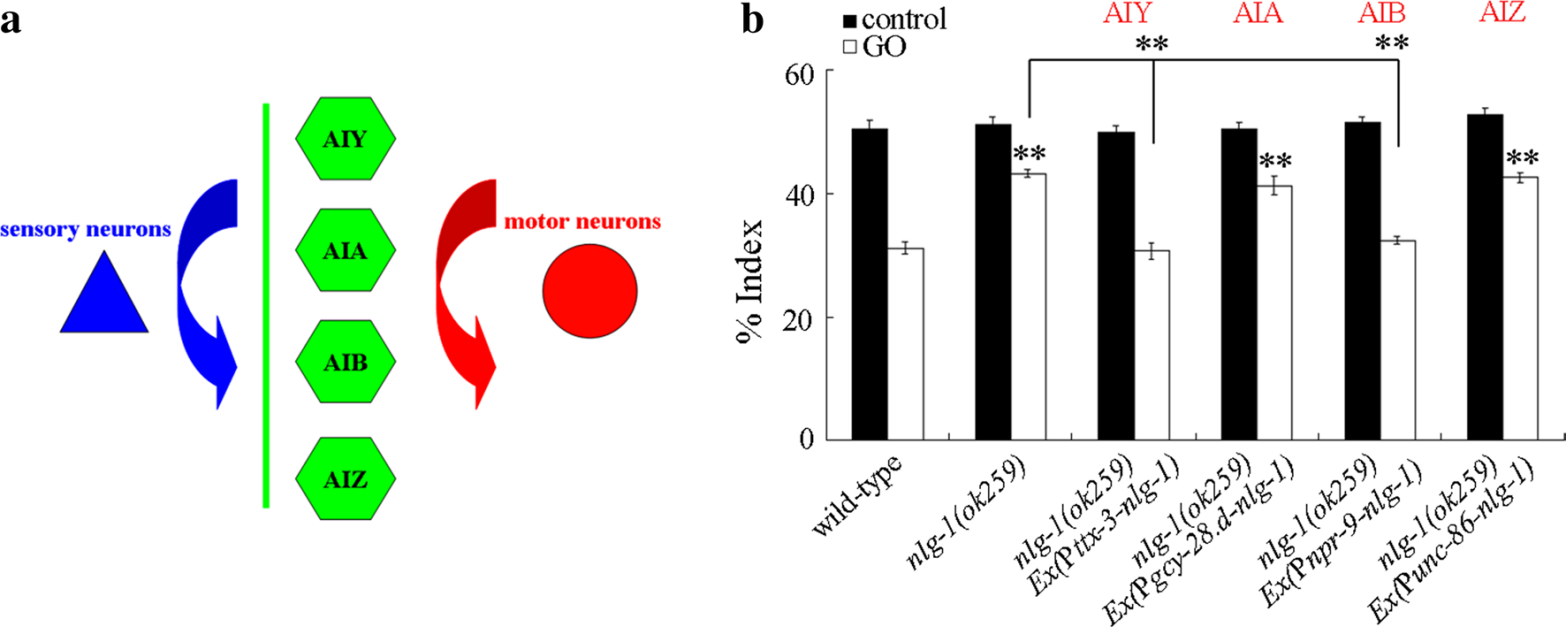
Identification of interneurons required for the aversive response of nematodes to GO. **a** A diagram showing the association of interneurons with sensory neurons and motor neurons. **b** Neuron-specific activity of NLG-1 in the regulation of aversive behavior of nematodes to GO (100 mg/L). Control, without GO treatment. Bars represent mean ± SD. **P < 0.01 vs wild-type (if not specially indicated)

In *C. elegans*, AIY, AIA, AIB, and AIZ interneurons are main classes of integrating neurons between sensory neurons and motor neurons (Fig. 4a) [39]. After 90 min treatment, we found that expression of *nlg*-*1* in AIA interneurons or AIZ interneurons could not rescue the deficit in aversive behavior to GO (100 mg/L) in *nlg*-*1*(*ok259*) mutant nematodes (Fig. 4b). In contrast, after 90 min treatment, neuron-specific expression of *nlg*-*1* in AIY interneurons or AIB interneurons could significantly decrease the index of aversive behavior to GO (100 mg/L) in *nlg*-*1*(*ok259*) mutant nematodes (Fig. 4b). Therefore, neuron-specific expression of *nlg*-*1* in AIY interneurons or AIB interneurons can rescue the deficit in aversive behavior to GO in *nlg*-*1*(*ok259*) mutant nematodes.

### Neuron-specific activity of NLG-1 in the regulation of GO toxicity

**Fig. 5:**
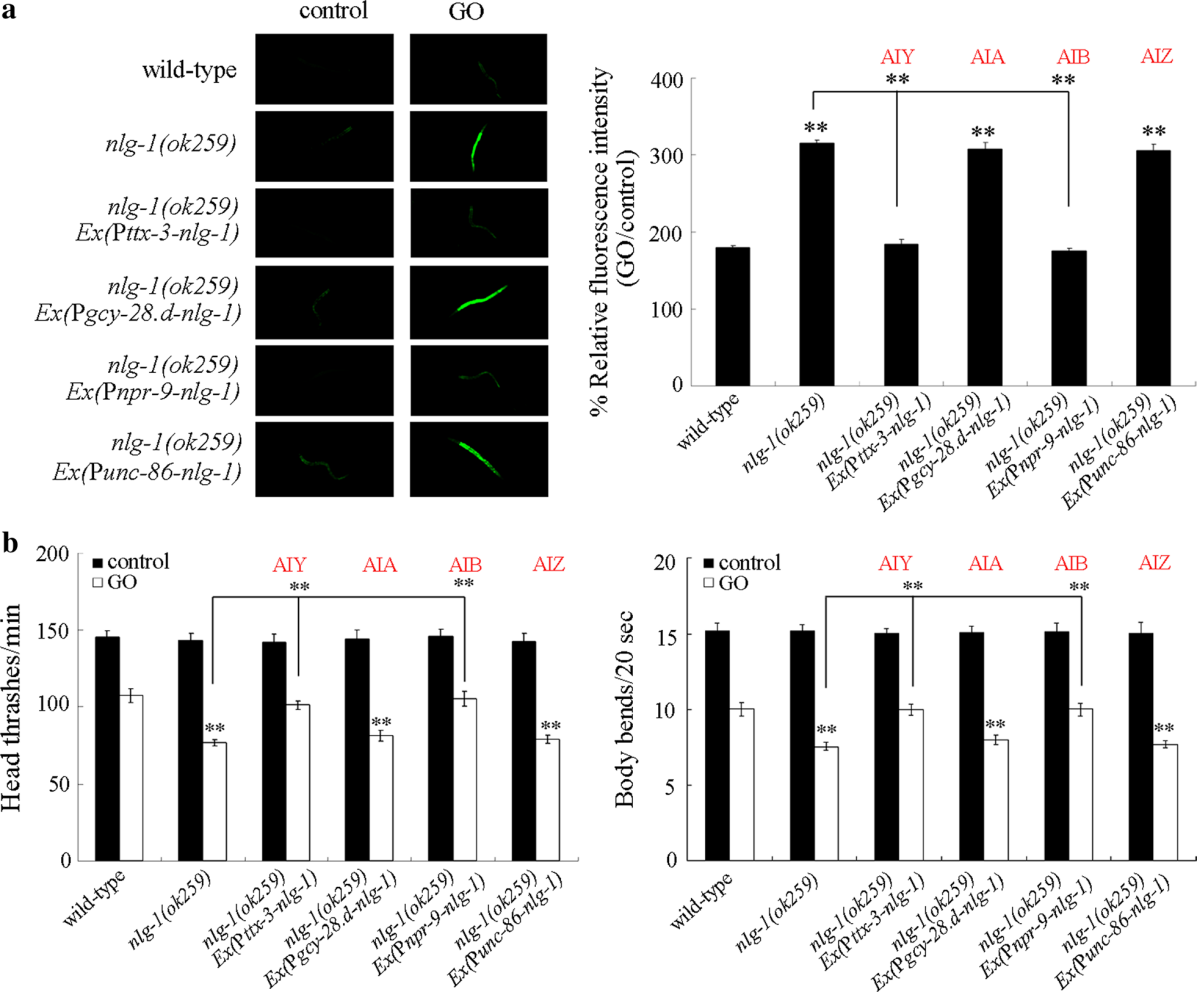
Neuron-specific activity of NLG-1 in the regulation of GO toxicity. **a** Neuron-specific activity of NLG-1 in the regulation of GO toxicity in inducing intestinal ROS production. **b** Neuron-specific activity of NLG-1 in the regulation of GO toxicity in decreasing locomotion behavior. GO exposure concentration was 10 mg/L. Prolonged exposure was performed from L1-larvae to young adults. Bars represent mean ± SD. **P < 0.01 vs wild-type (if not specially indicated)

We further determined the roles of these four classes of interneurons in the regulation of GO toxicity. Using intestinal ROS production and locomotion behavior as the toxicity assessment endpoints, we observed that expression of *nlg*-*1* in AIA interneurons or AIZ interneurons could not obviously affect the GO toxicity in inducing intestinal ROS production and in decreasing locomotion behavior in *nlg*-*1*(*ok259*) mutant nematodes (Fig. 5). In contrast, expression of *nlg*-*1* in AIY interneurons or AIB interneurons significantly suppress the GO toxicity in inducing intestinal ROS production and in decreasing locomotion behavior in *nlg*-*1*(*ok259*) mutant nematodes (Fig. 5). Therefore, both AIY interneurons and AIB interneurons are also required for the regulation of GO toxicity in nematodes.

## Discussion

*Caenorhabditis elegans* is a useful model for toxicity assessment of environmental toxicants [11, 12]. In this study, using the in vivo assay system of *C. elegans*, we observed the obvious aversive behavior of nematodes to GO particles (Fig. 2). Moreover, we also observed the significant aversive behavior of nematodes to TiO_2_-nanoparticles (TiO_2_-NPs, 10 nm), Al_2_O_3_-NPs (60 nm), multi-walled carbon nanotubes (MWCNTs), or thiolated GO (GO-SH) in nematodes (Additional file 1: Fig. S1). The detailed information on the physicochemical properties of examined TiO_2_-NPs, Al_2_O_3_-NPs, MWCNTs, or GO-SH is available in the references [40–43]. These observations imply that the nematodes have the potential ability to avoid the environmental ENMs once percept the existence of ENMs in the environment. This observed aversive response enables the environmental animals a protection mechanism to reduce the possible toxicity of environmental ENMs. Nevertheless, the examined GO at environmentally relevant concentrations may not be able to induce the aversive behavior of nematodes after 90 min treatment.

In *C. elegans*, it has been shown that NLG-1/neuroligin is required for the control of synaptic function, a subset of sensory behaviors and sensory processing, longevity, and oxidative stress or stress response [23, 44–47]. In this study, we further found a novel function of NLG-1 in the regulation of aversive behavior to GO. Loss-of-function mutation of *nlg*-*1* significantly suppress the aversive behavior to GO (Fig. 3), implying that NLG-1/neuroligin is required for the formation of normal aversive behavior to GO. Because the neuroligins act as a link to connect pre- and post-synaptic neurons in organisms [24, 25], our results suggest that a certain neuronal circuit connected by NLG-1/neuroligins may exist to regulate the aversive behavior of nematodes to GO.

**Fig. 6:**
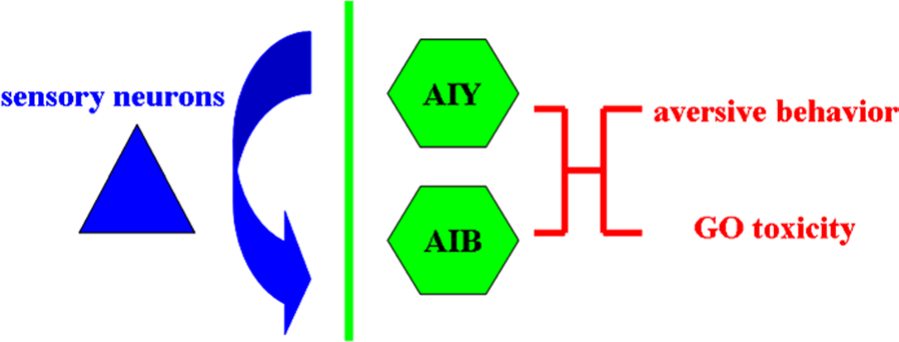
A diagram showing the functions of AIY and AIB interneurons in the regulation of aversive behavior to GO and GO toxicity

We further provide the evidence to demonstrate the crucial function of interneurons in the regulation of aversive behavior to GO. Among the main classes of interneurons with the function to integrate sensory neurons with motor neurons [39], we observed that only expression of *nlg*-*1* in AIY interneurons or AIB interneurons could recover the deficits in aversive behavior to GO in *nlg*-*1* mutant nematodes (Fig. 4). In contrast, the neuron-specific activity of *nlg*-*1* in AIA or AIZ interneurons was not required for the function of NLG-1 in the regulation of aversive behavior to GO (Fig. 4). These results imply that AIY and AIB interneurons are involved in the regulation of aversive behavior to GO (Fig. 6). Because the integration between sensory neurons and motor neurons by interneurons is conserved between the nematodes and the mammals or the human, our results further imply the crucial role of interneurons in the perception of toxic ENMs in mammals or in human.

In *C. elegans*, genetic or laser ablation of AIY interneurons caused the abnormal spontaneous reversal rate, odorant chemotaxis, and salt chemotaxis [48–50]. Additionally, genetic or laser ablation of AIB interneurons caused the abnormal odorant chemotaxis, and salt chemotaxis [49, 50]. Our results imply the novel function of AIY and AIB interneurons in the control of aversive behavior of nematodes to environmental toxicants. In *C. elegans*, it was repotted that genetic or laser ablation of AIY interneurons can enhance the spontaneous reversal rate [48]. Therefore, the identified interneurons may not only mediate a certain neuronal circuit to regulate the aversive behavior to GO, but also be able to directly participate in the regulation of aversive behavior themselves.

Our previous study has identified the neuron-specific activity of *nlg*-*1* in the AIY interneurons in the regulation of GO toxicity [23]. In this study, we further found the neuron-specific activity of *nlg*-*1* in the AIB interneurons in the regulation of GO toxicity (Fig. 5). These results all imply the crucial function of NLG-1 in interneurons in the regulation of GO toxicity (Fig. 6). In *C. elegans*, AIY interneurons act as the output of ASE, AWC, AFD, and AWA sensory neurons, and AIB interneurons act as the output of ASE, AWC, ASI, ASH, ASK, ADL, AFD, and ASG sensory neurons [38]. In *C. elegans*, the neurexin gene is *nrx*-*1.* The further examination of neuron-specific activities of NRX-1 will be helpful for final identification of neuronal circuit required for the control of aversive behavior to GO in nematodes.

## Conclusions

In this study, we investigated the aversive response of animals to GO using the in vivo assay system of *C. elegans*. We observed the obvious aversive behavior of nematodes to GO at concentrations more than 50 mg/L. In nematodes, mutation of *nlg*-*1* encoding a neuroligin disrupted this aversive behavior to GO. Using *nlg*-*1* mutant as a genetic tool, we identified the AIY and AIB interneurons to be required for the regulation of aversive behavior to GO based on a series of rescue assays. Our results provide the important neuronal and molecular basis for the aversive response of animals to GO. Moreover, we found that both the AIY interneurons and the AIB interneurons were also required for the regulation of GO toxicity in nematodes.

## Additional file

**Additional file 1.** Additional table and figure.
