# Supplementary material for: Identification of interneurons required for the aversive response of Caenorhabditis elegans to graphene oxide
Source: J Nanobiotechnology. 2018 Apr 27;16:45. doi: 10.1186/s12951-018-0373-y (PMC5921546; doi:10.1186/s12951-018-0373-y)
Supplement: Supplementary file 1 — Additional file 1. Additional table and figure. [file 12951_2018_373_MOESM1_ESM.doc]

**Additional Information:**

**
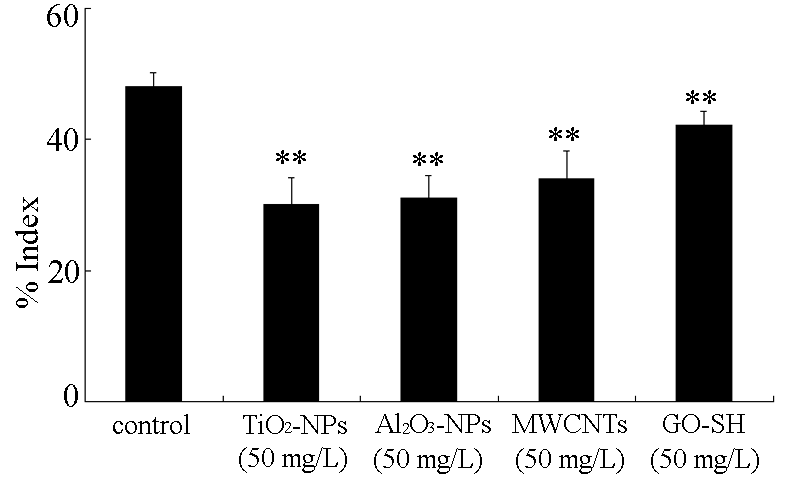
**

**Fig. S1** Aversive behavior of wild-type nematodes to TiO_2_-NPs, Al_2_O_3_-NPs, MWCNTs, or GO-SH. Control, without treatment. Bars represent means ± SD. ^**^*P* < 0.01 *vs* control.

**Table S1.** Primer information for vector constructions

| Gene | Forward primer (5’-3’) | Reverse primer(5’-3’) |
| --- | --- | --- |
| P*ttx-3* | ATCTCTAGAATATGCACCCCGCTGACA | TGCGGATCCTTGAAAAGTAGGAAGCAT |
| P*gcy-28.d* | GCGCTGCAGTGTCTGTGATTAAAAAAA | ATAGGATCCTTCGCACTCATCTCACCA |
| P*npr-9* | CGCCTGCAGGTTTTGGAAATGTTTAGA | ATAGGATCCGGGTGAACGTGTATTTAC |
| P*unc-86* | CGCAAGCTTAGCACTTGTAATTTCTTT | ATAGGATCCGAAAATATTTTGGGATCA |
| *nlg-1/C40C9.5e* | ATACCCGGGATGGAACGCATTTATCTT | GTGGAATTCATGCGAAAATAGTATACT |
